# Nonlinear Analysis of Electrocardiography Signals for Atrial Fibrillation

**DOI:** 10.1155/2013/509784

**Published:** 2013-05-13

**Authors:** Necmettin Sezgin

**Affiliations:** Department of Electrical and Electronics Engineering, Faculty of Architecture and Engineering, Batman University, 72060 Batman, Turkey

## Abstract

This paper aims to analyze the electrocardiography (ECG) signals for patient with atrial fibrillation (AF) by using bispectrum and extreme learning machine (ELM). AF is the most common irregular heart beat disease which may cause many cardiac diseases as well. Bispectral analysis was used to extract the nonlinear information in the ECG signals. The bispectral features of each ECG episode were determined and fed to the ELM classifier. The classification accuracy of ELM to distinguish nonterminating, terminating AF, and terminating immediately AF was 96.25%. In this study, the normal ECG signal was also compared with AF ECG signal due to the nonlinearity which was determined by bispectrum. The classification result of ELM was 99.15% to distinguish AF ECGs from normal ECGs.

## 1. Introduction

Electrocardiography (ECG) signals are electrical activity of the heart detected by electrodes that were attached to the surface of the skin and were recorded by a device with noninvasive method. The ECG is the best way to measure and present abnormal rhythms of the heart. Atrial fibrillation (AF) is the most irregular heart beat disease which may cause many cardiac diseases as well. During AF the nonlinearity of the heart increases and the analysis should be considered in nonlinear situations. For this reason, bispectral analysis which detects and reveals the nonlinearity of a signal was considered. A detailed description about bispectral analysis can be found in the next section. In the present study the bispectral analysis was implemented, and phase relations that are called quadratic phase coupling (QPC) of ECG signals were extracted. The energy, minimum, maximum, mean, and standard deviation of QPCs were determined and fed to classifiers in order to classify AF ECGs and separate AF ECGs from normal ECGs. The AF ECGs were classified in three groups: nonterminating AF (N), terminating AF (S), and terminating immediately AF (T).

In this study, the extreme learning machine (ELM) was performed as classifier for the classification and diagnosing of AF. The ELM is a feedforward neural network which has single hidden layer. In the ELM classifier, the weights between input and hidden layers and hidden node's biases are assigned randomly while the weights between hidden and output layers are determined analytically [1]. The most important feature of this technique is that it converges to the desired error point very fast. The accuracies of ELM are 96.25% and 99.15% for classification and diagnosing of AF, respectively. For a comparison the same data was trained and tested with artificial neural network (ANN) and support vector machine (SVM). However, the best performance was obtained by ELM classifier. The proposed method is thought to be serviced in clinics so that the cardiologists can classify and diagnose AF very swiftly with acceptable accuracies.

## 2. Materials and Methods

### 2.1. Data Recordings

The AF data was provided from Holter recordings in PhysioNet for a total of 80 recordings. The AF data has three groups: nonterminating AF (N—defined as AF that was not terminated for the duration at least an hour following the segment—25 recordings), terminating AF (S—defined as AF that terminates one minute after the end of the record—20 recordings), and terminating immediately AF (T—defined as AF that terminates within one second—35 recordings). And 50 normal ECG recordings were obtained from 15 healthy volunteers at Dicle University. Each data is one minute in length and has sampling frequency of 128 Hz.

### 2.2. Bispectrum Analysis

Bispectrum analysis is a statistical process which measures the phase degree of coupling present in a time domain signal [2, 3]. The Fourier transform of the second-order cumulant, that is, the autocorrelation function, is the traditional power spectrum. The Fourier transform of *R*(*τ*_1_, *τ*_2_) (third-order cumulant function) is called the bispectrum or bispectral density. They fall in the category of higher order spectra (HOS) or polyspectra and provide supplementary information to the power spectrum. One of the disadvantages of using power spectrum is that it suppresses phase information in the signal. However, a third-order spectrum or bispectrum preserves phase information. Since ECG contains substantial clues about cardiac diseases it could be well to take out phase information additionally.

Bispectral analysis has found success in the area of identifying phase relations of signals between different frequency bands [4]. In contrast to power spectrum estimation, bispectrum estimation reveals the non-Gaussian and nonlinear information. This allows the detection and characterization of nonlinear mechanisms which produce time series through phase relations of their harmonic components.

Let *x*(*n*) be a discrete, stationary, zero-mean random process, and its third-order cumulant sequence *R*(*τ*_1_, *τ*_2_) will be identical to its third-moment sequence. Hence,

(1) $$\begin{array}{c} R\left(\tau_{1},\tau_{2}\right)=E\left\{x\left(k\right)x\left(k+\tau_{1}\right)x\left(k+\tau_{2}\right)\right\}, \end{array}$$

where *E*{ } denotes the expectation operation.

Transforming the third-order cumulant into frequency domain yields the bispectrum:

(2) $$\begin{array}{c} B\left(\omega_{1},\omega_{2}\right)=\sum_{\tau_{1}=-\infty \;}^{\infty }\sum_{\tau_{2}=-\infty }^{\infty }R\left(\tau_{1},\tau_{2}\right)W\left(\tau_{1},\tau_{2}\right)e^{-j(\omega_{1}\tau_{1}+\omega_{2}\tau_{2})}. \end{array}$$

In (2) *W*(*τ*_1_, *τ*_2_) represents a two-dimensional window function which is employed in order to reduce the variance of the bispectrum estimation. Equation (2) is equivalently expressed as an average over the Fourier transform *X*(*ω*) of *x*(*n*):

(3) $$\begin{array}{c} B\left(\omega_{1},\omega_{2}\right)=E\left\{X\left(\omega_{1}\right)X\left(\omega_{2}\right)X^{*}\left(\omega_{1}+\omega_{2}\right)\right\}, \end{array}$$

where *B*(*ω*_1_, *ω*_2_) is the bispectrum of *x*(*n*). In general *B*(*ω*_1_, *ω*_2_) is complex, and a sufficient condition for its existence is that *R*(*τ*_1_, *τ*_2_) is absolutely summable.

*B*(*ω*_1_, *ω*_2_) is a symmetric function, such that a triangular region 0 ≤ *ω*_2_ ≤ *ω*_1_, *ω*_1_ + *ω*_2_ ≤ *π* could completely describe the whole bispectrum. A peak observed in the triangular region indicates that the energy component at frequency *ω*_1_ + *ω*_2_ is produced, likely due to the quadratic nonlinearity dependence which was called quadratic phase coupling (QPC), at the bifrequency (*ω*_1_, *ω*_2_) [5]. On the contrary, a flat bispectrum at the two frequency components *ω*_1_ and *ω*_2_ suggests no such activities. Consequently, phase coupled components contribute extensively to the third-order cumulant sequence of a process. This unique capability of bispectral analysis becomes a useful tool to detect and quantify the possible existence of QPCs in the ECG signals of AF patient.

The bispectral analysis was performed based on the direct method that uses fast Fourier transform (FFT) algorithm to reduce the computation time for estimating the bispectrum [6].

### 2.3. Extreme Learning Machine

The ELM is a feedforward neural network having only one hidden layer. The weights between input layer and hidden layer are selected randomly while the weights between hidden layer and output layer are determined analytically. In the ELM algorithm the activation functions such as sigmoid, sine, Gaussian, and hard limit are used in the hidden layer; however, the linear activation function is used in the output layer. The nonderivative and discrete activation functions can be used in the ELM [7].

In the ELM algorithm, since the input weights and biases are chosen randomly and the output weights are determined analytically, the network converges promptly. So, the ELM has better performance and is faster in some situation comparing with traditional methods [1, 8].

For an input data set, *X* = {**x**_*k*_}, let the desired outcome data from the network be *Y* = {**y**_*j*_} and, the real outcome of the network be *O* = {**o**_*k*_}, where *k* ∈ [1, *M*] represents the number of consequent input/output vectors. The mathematical description of the network having *M* neuron in the hidden layer can be expressed as [7]

(4) $$\begin{array}{c} \sum_{i=1}^{M}\beta_{i}g\left(w_{i}x_{k}+b_{i}\right)=o_{k},\;k=1,2,3,\ldots ,N, \end{array}$$

where **x**_*k*_ = [*x*_*k*1_, *x*_*k*2_, *x*_*k*3_,…, *x*_*kn*_]^*T*^ and **o**_*k*_ = [*o*_*k*1_, *o*_*k*2_, *o*_*k*3_,…, *o*_*km*_]^*T*^ are the input and output vectors for the *k*th trial, respectively, **w**_*i*_ = [*w*_*i*1_, *w*_*i*2_, *w*_*i*3_,…, *w*_*in*_] are the weights between input nodes and *i*th hidden node biased by *b*_*i*_, *β*_*i*_ = [*β*_*i*1_, *β*_*i*2_,…, *β*_*im*_] are weights between hidden nodes and *i*th output node, and *g*(·) is the activation function [1].

In this algorithm, the goal is to tune the weights *β* in accordance with minimization of cost function defined as the total error square at the output of the network.

For a network free from error, then (4) can be expressed in the matrix form as [1]

(5) $$\begin{array}{c} H\beta =Y, \end{array}$$

where **H**, *β*, and **Y** can be expressed as [7]

(6) $$\begin{array}{c} H={\left[\begin{array}{ccc} g\left(w_{1}x_{1}+b_{1}\right) & \cdots & g\left(w_{M}x_{1}+b_{1}\right)\\ \vdots & & \vdots \\ g\left(w_{1}x_{N}+b_{1}\right) & \cdots & g\left(w_{M}x_{N}+b_{M}\right) \end{array}\right]}_{M\times N} \end{array}$$

(7) $$\begin{array}{c} \beta ={\left[\begin{array}{ccc} \beta_{1} & \cdots & \beta_{M} \end{array}\right]}_{m\times M}^{T}, \end{array}$$

(8) $$\begin{array}{c} Y={\left[\begin{array}{ccc} y_{1} & \cdots & y_{N} \end{array}\right]}_{m\times N}^{T}. \end{array}$$

**H** is the output matrix of the hidden layer, and **Y** is the actual output matrix.

Although all of learning algorithms had been designed to reach a zero error, it is not possible in practice due to finite training time and/or local minima. Usually the concentration is made toward a smallest possible error reached in a reasonable training time. Therefore, in applications as the error reaches an acceptable error then the training period of network is terminated. In this case, (5) can be modified to approximately describe the system as $H\hat{\beta }=Y$ or, conveniently, $\hat{\beta }=H^{†}Y$, where **H** ^†^ is the generalized inverse of matrix **H**, called Moore-Penrose matrix [9, 10]. Ultimately, the ELM algorithm can be summarized in three steps [1, 11].

- Generate the input weights, **w**_*i*_ = [*w*_*i*1_, *w*_*i*2_, *w*_*i*3_,…, *w*_*in*_], and hidden layer bias values *b*_*i*_ randomly.
- Determine the hidden layer output matrix **H** and its inverse **H** ^†^ in accordance with input data as in (6).
- Calculate the output weights $\hat{\beta }$, by using $\hat{\beta }=H^{†}Y$.

## 3. Results

**Figure 1:**
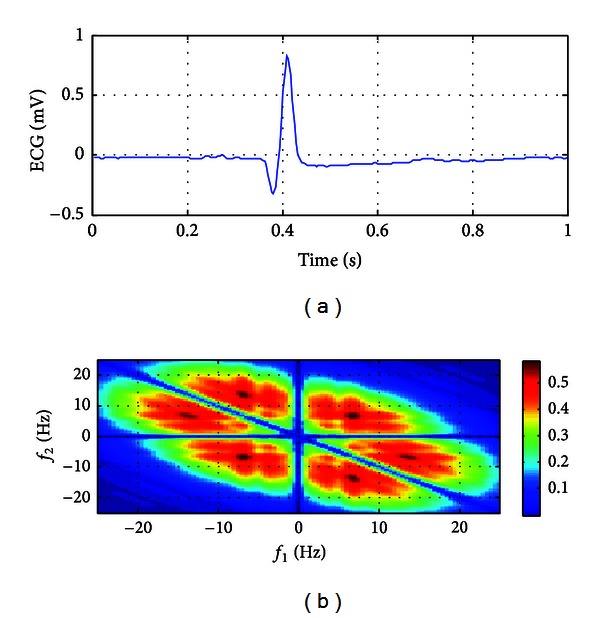
An ECG episode and its bispectrum for patient with nonterminating AF.

**Figure 2:**
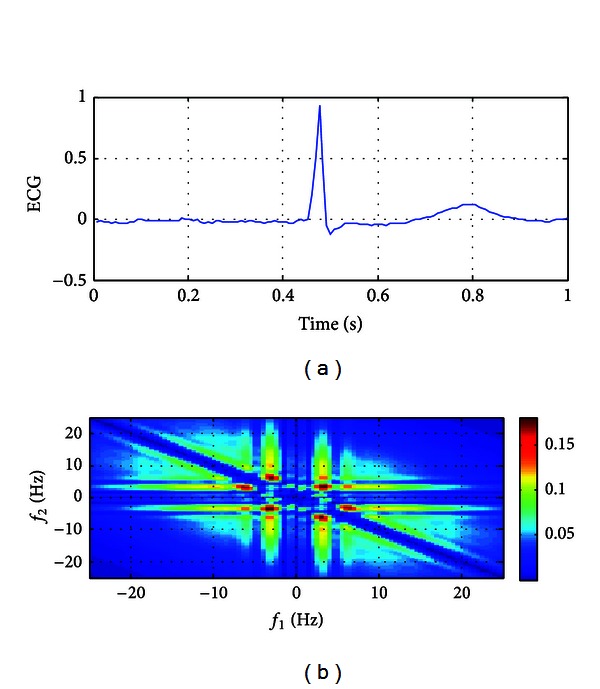
An ECG episode and its bispectrum for normal patient.

The ECG data of 80 AF recordings and 50 normal recordings were considered and analyzed. At first, all data were transformed in frequency domain with bispectral analysis. Then, the features such as energy, minimum, maximum, mean, and standard deviation of QPCs that were calculated from bispectral analysis were extracted. Lastly, these features were fed to input of the classifier. The ECG episodes of an AF patient and a normal patient of 1-second long and their corresponding bispectrum presentation are shown in Figures 1 and 2. As can be seen from figures, the bispectral density of AF ECG (Figure 1(b)) is higher than the bispectral density of normal ECG (Figure 2(b)). Furthermore, in the AF, the QPCs of ECG are distributed in wider range than QPCs of normal ECG in frequency domain. In Figure 1(b) the QPCs have been emanated in bifrequency range of 5–15 Hz; however, as seen in Figure 2(b), the QPCs are emerged at bifrequency of about 3 Hz.

The single hidden layer neural network, ELM, was trained and tested with data rate of 50%-50% both in classification and diagnosis of AF. In the ELM algorithm, the sigmoid activation function in the hidden layer and linear activation function in the output layer have been found to be much better by trial and error. The classification result of AF ECGs and diagnostic result of AF ECGs were 96.25% and 99.15%, respectively. Furthermore, the most used classifiers such as ANN and SVM were used instead of ELM and the overall results of them are presented in Table 1.

## 4. Discussion and Conclusions

In this study, classification of ECG signals which belong to AF terminating and benign patients was performed with ELM classifier. The bispectrum of each episode of ECG signals was analyzed, and energy, minimum, maximum, mean, and standard deviation of QPCs were determined and fed to input of the ELM. The performance accuracy was 96.25% in order to classify AF terminating groups and 99.15% in order to separate AF ECGs from normal ECGs. Furthermore, for a comparison, ANN and SVM classifiers were used for the same data, and lower accuracies were obtained by comparing with ELM. The overall results are shown in Table 1. The accuracy which obtained by the ELM is satisfactory to differentiate AF patients from normal patients. This simple and effective method may help to cardiologists in their final decisions to diagnose AF.

## Figures and Tables

**Table 1:** The performances of ANN, SVM, and ELM for classifying AF groups and separating AF ECGs from normal ECGs.

	Classifier	Training process time (sec)	Testing process time (sec)	Accuracy (%)
Classification of AF groups (N, T, S)	ANN	44.25	2.27	94.50
	SVM	1.75	0.13	90.15
	ELM	**0.03**	**0.005**	**96.25**

Separation of AF ECG from normal ECG	ANN	34.25	2.15	97.60
	SVM	2.30	0.12	92.35
	ELM	**0.03**	**0.005**	**99.15**
